# Doll Therapy Intervention Reduces Challenging Behaviours of Women with Dementia Living in Nursing Homes: Results from a Randomized Single-Blind Controlled Trial

**DOI:** 10.3390/jcm11216262

**Published:** 2022-10-24

**Authors:** Valentina Molteni, Roberta Vaccaro, Roberta Ballabio, Laura Ceppi, Marco Cantù, Rita B. Ardito, Mauro Adenzato, Barbara Poletti, Antonio Guaita, Rita Pezzati

**Affiliations:** 1 Dipartimento di Economia Aziendale e Socio Sanitaria (SUPSI), Centro Competenza Anziani, 6928 Manno, Switzerland; 2 GINCO Ticino Association, 6802 Monteceneri, Switzerland; 3 Golgi Cenci Foundation, 20081 Abbiategrasso, Italy; 4 School of Cognitive Therapy, 22100 Como, Italy; 5 Ente Ospedaliero Cantonale, Istituto di Medicina di Laboratorio (EOLAB), 6500 Bellinzona, Switzerland; 6 Department of Psychology, University of Turin, 10124 Turin, Italy; 7 Laboratory of Neuroscience, Department of Neurology, Istituto Auxologico Italiano, IRCCS, 20149 Milan, Italy

**Keywords:** attachment, biomarkers of stress, caregivers, challenging behaviours, dementia, doll therapy, non-pharmacological therapies

## Abstract

Background: Doll therapy (DT) is a non-pharmacological intervention for the treatment of the behavioural and psychological symptoms of dementia (BPSD). We designed a single-blind randomized controlled trial of the 30-day efficacy of DT in reducing the BPSD, professional caregivers’ distress and patients’ biomarkers of stress, and in improving the exploration and caregiving behaviours. Methods: We randomly assigned 134 women with moderate-to-severe dementia living in nursing homes (NHs) to a DT intervention (DTI, 67) or a sham intervention with a cube (SI, 67). Results: From the first to the 30th session, the DTI group showed a significant decrease in the Neuropsychiatric Inventory-NH (NPI-NH) total score and in the NPI-NH-Distress score compared to the SI group (both *p* < 0.001). We observed a greater interest in the doll than in the cube, a greater acceptance of a separation from the nurse among DTI participants, and caregiving and exploratory behaviours towards the doll. There were no differences between the groups in the stress biomarkers. Conclusions: Consistent with attachment theory, our findings support the 30-day efficacy of DT, as this non-pharmacological intervention promotes perceptions of security by creating a situation in which patients feel confident and engaged in a caregiving relationship with the doll and reduces the challenging behaviours that are stressful for professional caregivers.

## 1. Introduction

Non-pharmacological therapies (NPTs) to support people with dementia (PWD) are recommended as first-line treatments for the behavioural and psychological symptoms of dementia (BPSD) [1]. More than half of people with moderate to severe dementia have at least one BPSD, with apathy, anxiety, irritability, agitation, and depression being the most common [2,3]. Such challenging behaviours are associated with an increased caregiver burden [4] and a poor quality of life for both the patients and caregivers [5]. Doll therapy (DT) is an NPT for people in the advanced stages of dementia, with the aim of reducing challenging behaviours. Promising results have already been shown in the literature on the effectiveness of DT in promoting and maintaining the affective-relational dimension of attachment caregiving [6,7]. DT appears to improve verbal [8,9] and non-verbal communication, including eye contact and touch [10], exploration and caregiving behaviours [7], and a patient’s interaction with others [11,12]. Promising results have also been shown in relation to professional caregivers’ distress [13,14]. However, the available studies are mainly pilot or exploratory studies [7,13,15,16]. Randomized controlled trials with rigorous study designs and larger samples are critical for determining the effectiveness of DT implemented in long-term settings and for identifying the best practice for interventions.

Another area of interest is the explanatory model of the DT process. The concept of attachment, theorized by John Bowlby [17], has been applied to the relationship with the doll [7,18]. Attachment theory states that the human tendency to seek closeness and protection when feeling vulnerable or insecure is an expression of an innate motivational system. This theory is particularly relevant for PWD, as dementia often exposes patients to feelings of personal vulnerability and loss, as well as experiences of separation [19,20]. Miesen suggested that PWD in the advanced stages of the disease experience a constant loss of control and security, a situation that constitutes a constant trauma that the patient tries to cope with by seeking reassurance and protection from the persons present, thereby activating attachment behaviours: “this is a normal reaction to an abnormal situation” [21]. Due to the cognitive and psychological impairments caused by the disease, attachment is therefore often considered as an essential psychological need [22]. From this perspective, BPSD such as wandering, dysphoria, anxiety, agitation, and aggressiveness could be interpreted as attachment requests. A clinical observation of PWD interacting with the doll has shown that they treat the doll like a real baby and thus replace the requests for care and protection (with typical attachment behaviours such as vocalizations, gestures, and tears) with caregiving behaviours, such as reassuring and cradling the doll. Following this theory, we assume that DT restores a sense of calm and peace which leads to the cessation of requests for a proximity to caregivers expressed by BPSD [7,22]. In nursing home (NH) settings, PWD are often exposed to feelings of insecurity in situations of separation from professional caregivers. Understanding the attachment behaviours of PWD can shift caregivers’ attention from challenging behaviours to the relationship needs of PWD. In our study, we specifically addressed the training of professional caregivers to improve their knowledge about DT and their awareness of their relationship with PWD.

Blood pressure and heart rate are involved in the response to acute and chronic psychological stressors. Secretion of salivary cortisol is the final product of the activation of stress-response mechanisms, i.e., the hypothalamic-pituitary-adrenal axis [23]. Changes in the rate of its secretion have been associated with acute stress responses related to disease and cognitive impairment [24,25]. To our knowledge, there is limited data on the effectiveness of NPTs on biomarkers of stress in PWD, although encouraging results have recently been reported for mindfulness interventions for cognitive impairment [26]. To date, no studies have examined the effect of DT on biomarkers of stress in PWD.

Our primary aims were to add scientific data on the effectiveness of DT on the challenging behaviours of PWD and professional caregivers’ distress in nursing home settings on a large population sample. We intended to add data on the efficacy of DT on biomarkers of stress in PWD, an area for which scientific evidence is not yet available. Finally, our aim was to show that DT promotes feelings of security by creating a situation in which PWD feel confident and engaged in a caregiving relationship with the object in the absence of the nurse. We also expected an increase in the exploration and caregiving behaviours of the patients during the interaction with the doll. The secondary aim was to confirm the hypothesized stability of the PWDs previous attachment style (determined by the Adult Attachment Interview administered to the patients’ family caregivers), even in the advanced stages of dementia. For the purposes of the present work, we have not presented the results of the secondary aim.

## 2. Materials and Methods

### 2.1. Design and Participants

This study is a randomized, single-blind, controlled trial with two parallel arms designed to evaluate the 30-day effectiveness of the DT intervention (DTI) compared to the sham intervention (SI) in women with dementia living in a NH. An independent statistician performed the computer-based block randomization [27]. The participants were assigned using a concealed 1:1 randomization. The statistician generated the randomization sequence and forwarded it to the study coordinator who received the randomization to assign patients to either group A or B (block size: 2 × 2 = 4).

To ensure blinding, the psychologist who administered the primary outcome instruments did not know which arm the participants belonged to. Conversely, the enrolled participants and the nurses were not blinded with respect of the objects provided (doll or cube). The participants were female with moderate to severe dementia (corresponding to stages 4–7 of the Global Deterioration Scale) [28] who lived in 26 NHs of the Canton Ticino area (Switzerland), had at least one BPSD in addition to depression or apathy, had been admitted to a NH at least 3 months previously, and had no previous exposure to DT. Participants who met the inclusion criteria were considered for the present study regardless of the type of dementia and the presence of speech disorders. The exclusion criteria were: the male gender, presence of comorbid mental disorders (i.e., major depression, bipolar disorders, and schizophrenia), inability to sit comfortably in a chair or limitations in arm mobility, and presence of acute clinical conditions that interfered with participation in the study.

The study completion was on 9 December 2019.

### 2.2. Ethical Consideration and Consent to Participate

The study received approval from the Swiss Ethics Committees on Research Involving Humans (n. CE3140 BASEC2016–01992) before starting. The patients or their legal representatives (families) were asked to sign a written informed consent form for the use of their personal data and were given concise and comprehensive information about the research objectives and the methods of data processing.

### 2.3. Intervention

The intervention procedure has been described in detail elsewhere [29]. Participants attended daily DTI or SI sessions for a maximum of one hour, led by a trained professional caregiver. DTI encompasses the presentation of an empathy doll recreating the sensation of touching, looking at, and holding a child in one’s arms. This kind of dolls elicits emotional reactions and provides opportunities for pleasurable sensory experiences [30]. The SI group participated in similar daily sessions, but a non-anthropomorphic object was presented (i.e., a soft foam rubber cube covered with a coloured and velvety textile). The DTI or SI was discontinued after seven consecutive refusals.

During the 18 months that the NHs were involved in the research project, training was provided for professional caregivers. It consisted of two classroom lessons, each lasting for four hours, on DT procedures and ten monthly clinical supervisions, each lasting half an hour, on single-case discussions aimed at improving the quality of the caregiving relationship as a source of security according to attachment theory. The maximum number of participants among professional caregivers was 25 per group. Each training group was conducted by trained psychologists and nurses. According to the Guidelines for the use of dolls as a therapeutic tool (www.fightdementia.org.au), dolls were proposed to PWD after considering their personal history, any traumatic events, and their parenting style. During their training, professional caregivers were made aware of the importance of paying attention to how the PWD interact with the doll (i.e., whether or not they treat the doll like a real baby) and validating the meaning it has for the patient. PWD are free to leave or refuse the doll at any time if they do not enjoy holding it.

### 2.4. Primary Outcomes’ Measurements

#### 2.4.1. Patient’s BPSD

The difference in the patient’s BPSD was measured as the net change in the Neuropsychiatric Inventory-Nursing Home (NPI-NH) total score, pre- to post-intervention. We expected a significant reduction in the NPI-NH total score in the experimental group compared to the active control group. The NPI-NH was validated for interviewing the professional caregivers on twelve behavioural areas, including depression [31,32]. The frequency [from one (rarely, less than once a week) to four (very often, once, or more per day)] and the severity [from one (mild) to three (severe)] of each BPSD are reported. The domain scores (frequency × severity) are added into the total score, ranging from zero to 144, with higher scores indicating more severe symptoms.

#### 2.4.2. Professional Caregivers’ Distress

The difference in the professional caregivers’ distress related to the patient’s BPSD was measured as the net change in the NPI-NH Distress score, pre- to post-intervention [31,32]. We expected a significant reduction in the NPI-NH Distress score in the experimental group compared to the active control group. When a BPSD is reported as present, the associated distress is rated on a scale from zero (no distress) to five points (very severe distress). The domain scores are added into the Distress score, ranging from zero to 60, with higher scores indicating more caregivers’ distress.

#### 2.4.3. Patient’s Biomarkers of Stress

The decrease in patient stress was measured as a net change in the blood pressure (systolic and diastolic), heart rate, and salivary cortisol level [23,24,25,33]. We expected a significant difference in the biomarkers of stress between the DTI and SI groups. The saliva samples, blood pressure, and heart rate were collected immediately before and 15 min after the end of the intervention at the first and 30th session. The saliva samples were stored at an ambient temperature for 24 h and then processed immediately. A ratio between ‘immediately before’ versus ‘after 15 min’ was calculated for the first and 30th session: a ratio of one meant that the cortisol concentration, and thus the stress level (both low and high), had not changed (coded as irrelevant treatment); if the ratio was greater than one, the cortisol concentration, and thus the stress level, had decreased (coded as an effective treatment); and if the ratio was less than one, the cortisol concentration, and thus the stress level, had increased (coded as negative treatment).

#### 2.4.4. Patient’s Interaction with the Object

We expected an increase in the interaction with the object (doll), as the exploration and caregiving behaviours during the object presentation were recorded at the first and 30th session by a trained psychologist who filled an observational grid developed for the aims of the present study [29]. It includes four kinds of behavioural responses: i.e., i. object presentation; ii. separation from the nurse; iii. interaction with the object; and iv. separation from the object. We expected significant differences between the DTI and SI groups from the first to the 30th session.

### 2.5. Statistical Analysis

#### 2.5.1. Sample Size

A sample size of 64 subjects per group, for an analysis of covariance (ANCOVA) that includes the pre-intervention NPI-NH total score as a covariate, was computed using an estimated medium effect size (f = 0.25), an alpha level of 0.05, and a power of 0.8. Thereafter, a 10% attrition rate due to possible acute clinical conditions interfering with the participation in the study, or death, was considered. The power calculation was performed with the programme G*Power 3.1 [34].

#### 2.5.2. Analysis

The quantitative variables were reported as the means with standard deviations (SD); the qualitative variables as were reported as percentages. Independent sample *t*-tests (or Mann–Whitney tests for the variables not normally distributed) or Chi-square tests according to the variable characteristics (continuous or binary, respectively) were performed to compare the experimental and active control groups in terms of sociodemographic, clinical characteristics, NPI-NH total, and NPI-NH Distress net change. The primary outcomes were reported as the adjusted mean and standard error (SE). An analysis of covariance (ANCOVA) was applied to compare the effects of the two experimental conditions (the DTI or SI group) on the post-intervention NPI-NH total score and NPI-NH Distress score. The pre-intervention NPI-NH total score and psychotropic medications were used as covariates in these analyses. The effect sizes of the between-groups changes according to Cohen (1992) [35] were also reported. A *p*-value of less than 0.05 was considered significant (two-sided). All analyses were conducted using SPSS Statistics 20.0 (IBM SPSS Statistics for Windows, version 20.0 (IBM Corp. Released 2011, Armonk, NY, USA). A per-protocol analysis was conducted.

## 3. Results

### 3.1. Participants

**Figure 1:**
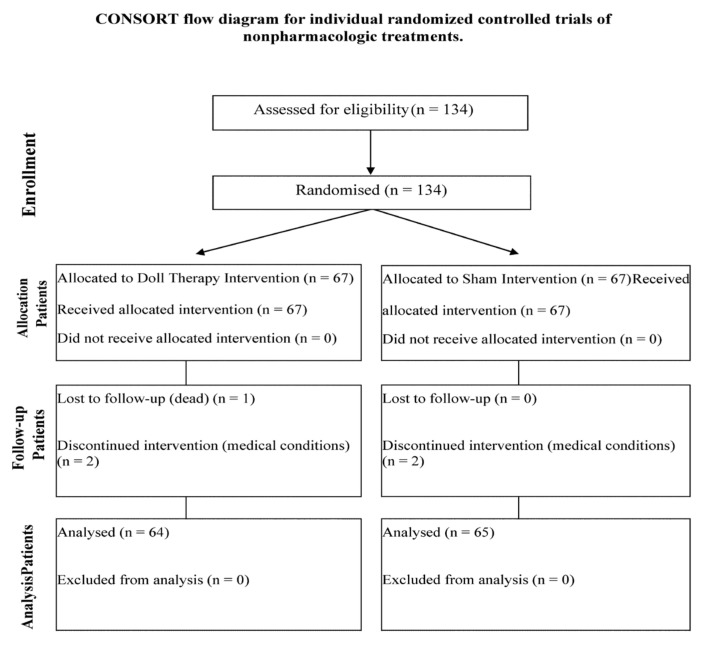
CONSORT flow diagram for individual randomized controlled trials of nonpharmacologic treatments.

There were 134 eligible participants. Four of them (two in the DTI group and two in the SI group) dropped out of the study and one in the DTI group died during the 30 days of the intervention (Figure 1). One hundred and twenty-nine participants completed the study, 64 in the DTI group and 65 in the SI group. The attrition rate was of 3.73%.

### 3.2. Baseline Characteristics

The baseline sociodemographic and clinical characteristics of the sample are shown in Table 1. No significant differences were found between the two groups in age (z = −1.276, *p* = 0.202), pre-intervention NPI-NH total score (z = −1.05; *p* = 0.292), NPI-NH Distress score (z = −1.86, *p* = 0.063), psychotropic medication (z = −0.217, *p* = 0.828), Mini Mental State Examination score (z = −1.79, *p* = 0.074), Global Deterioration Scale score (z = −1.146, *p* = 0.252), and diagnosis [Fisher’s exact test = 3.163, *p* = 0.552), indicating the effectiveness of the randomization procedure.

Primary outcomes.

### 3.3. Patient’s BPSD and Caregivers’ Distress

The DTI group showed a greater decrease in the NPI-NH net change [mean (SD): −11.9 (9.7)] compared to the SI group [mean (SD): −4.01 (7.1); z = −5.65; *p* < 0.001]. In particular, the DTI group showed a greater reduction in agitation compared to the SI group [mean (SD): −1.52 (3.17) and mean (SD): −0.36 (2.56), respectively; z = −2.796, *p* < 0.005], anxiety [mean (SD): −1.95 (2.99) and mean (SD): −0.23 (1.91), respectively; z = −3.929, *p* < 0.001], apathy [mean (SD): −1.31 (2.64) and mean (SD): −0.1 (1.7), respectively; z = −3.418, *p* < 0.001], irritability [mean (SD): −0.18 (2.06) and mean (SD): −0.23 (1.91), respectively; z = −2.679, *p* < 0.05], and wandering [mean (SD): −1.53 (2.69) and mean (SD): −0.4 (3.06); z = −2.231, *p* < 0.05]. The DTI group reported a significant decrease in NPI-NH Distress net change [mean (SD): −3.9 (3.9)], which was greater than that of the SI group [mean (SD): −1.07 (3.1).; z = −4.42; *p* < 0.001].

The results of the one-way ANCOVAs on the post-intervention BPSD of the PWD and caregiver’s distress are shown in Table 2. Levene’s test for equality of variances and normality checks were performed and the assumptions were met. The DTI group reported a significant greater decrease in the post-intervention NPI-NH total score with respect to the SI group, with a medium effect size. The DTI group reported a significant decrease in the post-intervention NPI-NH Distress score, greater than that in the SI group, with a medium effect size.

### 3.4. Patient’s Biomarkers of Stress

The differences between the DTI and SI groups in the systolic and diastolic pressure ratio and heart rate ratio at the first and 30th session of the intervention can be seen in Table 3. At the first session, there were 64 subjects in the DTI group and 62 in the SI group, while at the 30th session, 62 subjects in the DTI group and 60 subjects in the SI group were retained for analysis. Based on the results of the Mann–Whitney test, there was a trend towards significant differences only in the heart rate ratio at the 30th session, with the SI group having a lower heart rate ratio (mean rank = 56.13, *n* = 61) compared to the DTI group [mean rank = 68.67, *n* = 63; z = −1.946; *p* = 0.052]. As shown in Table 4, the complete cortisol concentration data were obtained for 45 participants (DTI group = 19 and SI group = 26). No significant differences between the two groups emerged in the stress level from the first to the 30th session of intervention.

### 3.5. Patient’s Interaction with the Object

#### 3.5.1. Object Presentation

The doll was accepted more often [mean (SD) = 23.09 (6.41)] than the cube [mean (SD) = 19.68 (7.93); (z = −2.339, *p* = 0.019); *p* < 0.005]. During the object presentation, in the DTI group (*n* = 62), among the 39 participants with a gaze direction “toward the nurse” in the first session, 15 directed their gaze “toward the object and the environment” in the last session (chi-square = 4.147, *p* < 0.05); whereas in the SI group (*n* = 57), among the 42 participants with a gaze direction “toward the nurse” in the first session, 37 subjects maintained their gaze on the nurse in the last session (chi-square = 17.094, *p* < 0.001).

#### 3.5.2. Separation from the Nurse

During the separation from the nurse, after the presentation of the object, among 11 participants in the DTI group (*n* = 61) who expressed “complain and worry” in the first session, 7 accepted the separation, 2 showed a “lack of interest”, and 2 continued to complain in the last session (Fisher’s exact test = 20.431, *p* < 0.001). In the SI group (*n* = 60), among 12 participants who expressed “complain and worry” in the first session, 7 continued to complain in the last session (Fisher’s exact test = 27.718, *p* < 0.001).

During the last session, caregiving behaviours were showed by 34 out of 43 participants in the DTI group (*n* = 63) who “accepted separation from the nurse”, by 5 out of 9 participants who expressed “complain and worry”, and only by 2 out of the 11 participants with a “lack of interest” in the separation from the nurse (Fisher’s exact test = 14.094, *p* < 0.001). Similarly, during the last session, exploration behaviours were present among 40 out of 43 participants in the DTI group (*n* = 63) who “accepted separation from the nurse”, 8 out of 9 participants who expressed “complain and worry”, and 7 out of 11 participants with a “lack of interest” in the separation from the nurse (Fisher’s exact test = 5.831, *p* < 0.05).

#### 3.5.3. Interaction with the Object

During the interaction with the object, the number of exploratory behaviours (e.g., touching, smelling, and looking at the object) did not differ in both groups during the first [DTI group: mean (SD) = 2.68 (0.8); SI group: mean (SD) = 2.72 (1); t = −0.218, *p* = 0.828] and the last session [DTI group: mean (SD) = 2.45 (0.93); SI group: mean (SD) = 2.47 (0.87); t = −0.091, *p* = 0.927]. Among 39 participants in the DTI group (*n* = 64) showing caregiving behaviours (e.g., caressing, cradling, talking, and smiling) in the first session, 6 did not show them in the last session, but among 25 participants not showing caregiving behaviours, 8 started to care for their dolls (chi-square = 18.319, *p* < 0.001). Of the participants in the SI group (*n* = 60), as expected, no one showed caregiving behaviours in the first session, but one participant started to care for her cube in the last session.

#### 3.5.4. Separation from the Object

During the separation from the object, in the DTI group (*n* = 64), “putting the object aside” did not differ from the first to the last session (chi-square = 0.755, *p* > 0.05). Of the 20 participants in the SI group (*n* = 63) who put the cube aside in the first session, 10 kept the cube with them in the last session (chi-square = 4.506, *p* < 0.05).

## 4. Discussion

The present study demonstrated the 30-day efficacy of DTI compared to SI, resulting in a greater improvement in the BPSD and caregivers’ distress. DTI produced significant reductions in agitation, anxiety, wandering, apathy, and irritability. This is of pivotal importance as BPSD affect the presentation and progression of the disease, accounting the most difficulties for PWD, their families, and the professional caregivers. These improvements in BPSD are clinically important because BPSD are associated with a greater functional impairment, accelerated cognitive decline, poorer quality of life, increased caregivers’ burden, higher mortality, and more neuropathological markers of dementia [36]. Our observation of DTI has shown that PWD show fewer signs of emotional discomfort (i.e., frustration and agitation), are more stimulated, communicative, and friendly with others.

As previously reported, the evidence base for medications currently used to treat BPSD is limited or their efficacy is moderate, and there is a risk of adverse events, including mortality [37]. Our results are from a randomized controlled trial with a large sample size in a long-term setting, which support previous limited evidence of the therapeutic effect of DT [6,7,13,14,15,38] and confirms that DT can be considered as a first-line treatment for targeting challenging behaviours in women with dementia living in a NH. As Losada-Baltar and Jiménez-Gonzalo (2021) noted [39], the availability of evidence-based treatments in applied, real-world contexts helps us translate evidence into practice. In addition, DT significantly reduced the perceived professional caregivers’ distress, contributing to a higher quality of personalized care for the PWD. In line with a recent systematic review [40], this beneficial outcome could be related to the use of multiple implementation strategies, i.e., the provision of the intervention as part of staff’s usual duties, training of professional caregivers aimed at understanding the mechanisms of the intervention, and supervisions aimed at providing continuous support.

The risk of infantilization and deception has been addressed in DT [41,42]. In line with the previous literature, we considered training professional caregivers, improving their knowledge, and letting them experience the positive effects of DT as key points to overcome these risks [8,43]. In our view, the benefits of DT fulfil the bioethical concepts of beneficence (i.e., DT promotes well-being) and respect for autonomy (i.e., PWD can exercise their right to engage with dolls if they wish).

To our knowledge, DT has no potential negative effects in the case of grief or infertility experienced during one’s life, since the doll is not experienced as one’s own child, but as the child of a nurse. The DTI therefore does not reactivate past memories of grief, but responds to the PWDs current need for care, which can no longer be exercised in the context of the nursing home.

DTI offers relevant advantages in terms of costs-effectiveness: unlike other nonpharmacological treatments (e.g., pets, music, or art therapy), it does not necessarily require the presence of a skilled therapist but can be carried out within 24 h by nursing home staff (nurses, assistants) if they are properly trained and supervised. As previously described [7,29], PWD take care of the dolls even in the absence of a professional caregiver.

We did not find significant effects of DTI on the biomarkers of stress, i.e., the blood pressure, heart rate, and salivary cortisol levels. Since we considered dementia to be a condition of chronic stress, we expected a beneficial effect of DT due to improvement in BPSD. However, a recent study reported that the reduction in systolic BP is more related to a proximity to death and sex than dementia or age [44]. The null results on cortisol levels might be related to the insufficient salivation of the participants, which we found when collecting the saliva samples, so that only a small proportion of the samples were analysed. Furthermore, the data showed a high individual variability of the cortisol levels.

In addition to the reduction in BPSD, we found increased behaviours related to the relationship with the environment and professional caregivers. We observed a great interest in the doll, as the PWD directed their gaze more often to the doll than to the cube. We found a significant increase in the acceptance of or disinterest in being separated from the nurse among the DTI individuals who initially expressed worries and complains. Finally, we observed the presence of caring and exploratory behaviours towards the doll in the DTI individuals who accepted the separation from the nurse. Consistent with the theoretical framework of attachment [21,45,46], these findings confirmed the hypothesis that the doll facilitates perceptions of security by creating a situation in which PWD feel confident and engaged in a caregiving relationship with the object in the absence of the nurse.

### Strengths and Limitations

To our knowledge, the present study is the first RCT on the effectiveness of DT with a large sample size in a long-term setting. Our findings thus enable us to identify the best practice for DTI with PWD and contribute to widespread DT. Another strength is the provision of training and supervision for professional caregivers to consolidate the procedures, and to have an ethical approach of the staff and to motivate them, as recommended by Cohen-Mansfield [47].

We acknowledge that this study has some limitations. First, the exclusion of men limits the generalizability of the results. As reported by the Department of Health and Sociality of the Canton Ticino (source: Statistica intra-muros, SOMED, Dipartimento della sanità e della socialità, Unità statistiche sanitarie, Bellinzona), 71.6% of PWD living in the NH of the Canton Ticino are women. Therefore, further research is needed to demonstrate the feasibility of DT in men. Secondly, the individual variability of salivary cortisol levels should be kept under control by estimating the individual levels at the baseline, i.e., by collecting the samples within 24 h before the start of the treatment. In addition, the scarce salivation of PWD made the collection of saliva samples much more difficult. Finally, we did not include a medium- to long-term follow-up on the effectiveness of the DT. However, professional caregivers administered DTI for as long as they evaluated it to be beneficial for PWD. Further research is needed to determine the medium- and long-term effects of DT in NHs.

## 5. Conclusions

The present study demonstrated the 30-day efficacy of DTI compared to SI and resulted in a greater improvement in the BPSD and caregivers’ distress. DT can be considered as a first-line treatment for challenging the behaviours of women with dementia living in NHs. However, further research is needed to determine the medium- and long-term effects of DT in NHs. Consistent with the theoretical framework of attachment, DT increases one’s perceptions of security by creating a situation in which PWD feel confident and engaged in a caregiving relationship with the doll and reduces the attachment behaviours that burden the professional caregiver. These benefits are not only a function of the tool used (the doll), but also of the relationship established between PWD and professional caregivers who are appropriately trained and aware of their role as an attachment figure.

## Figures and Tables

**Table 1:** Baseline sociodemographic and clinical characteristics by groups.

	DTI (*n* = 64)	SI (*n* = 65)
Age, mean (SD)	86.9 (5.9)	88.4 (5.5)
Pre-intervention ^#^ NPI-NH total score (range 0–144), mean (SD)	33.84 (13.28)	31.55 (12.1)
Pre-intervention ^¥^ NPI-NH Distress score (range 0–60), mean (SD)	12.08 (5.4)	10.6 (5.04)
Pre-intervention psychotropic medications, mean (SD)	1.87 (1.11)	1.81 (1.08)
Mini Mental State Examination (range 1–30), mean (SD)	7.92 (4.95)	10.27 (5.32)
Global Deterioration Scale (range 1–7), median (iqr)	6 (5–7)	6 (5–7)
Diagnosis, *n* (%)		
Alzheimer	23 (35.9)	24 (36.9)
Vascular	2 (3.1)	4 (6.2)
Mixed	7 (10.7)	9 (13.8)
Other	8 (12.5)	3 (4.6)
Non specified dementia	24 (37.5)	25 (38.5)

Values denote mean with standard deviation (SD) in parenthesis or median with interquartile range (iqr) in parenthesis or frequency with percentages in parenthesis. # Neuropsychiatric Inventory-Nursing Home total score. ¥ Neuropsychiatric Inventory-Nursing Home–Distress.

**Table 2:** Efficacy of DTI (experimental group) versus SI (control group) on post-intervention NPI-NH total and Distress score.

	DTI (*n* = 64)		SI (*n* = 65)				
Post-Intervention	Adjusted Mean (SE)	95% CI	Adjusted Mean (SE)	95% CI	^¶^ F	*p*-Value	^§^ Partial Ƞ^2^
NPI-NH total score	21.067 (1.014)	19.061–23.073	28.508 (1.018)	26.492–30.523	43.433	<0.001	0.638
NPI-NH Distress score	7.612 (0.435)	6.751–8.473	10.093 (0.432)	9.237–10.948	40.970	<0.001	0.625

^¶^ F value derived from ANCOVA adjusting for pre-intervention NPI-NH total score and psychotropic medications. ^§^ Effect size relationship between the pre-intervention and post-intervention scores.

**Table 3:** Comparison between the experimental and control groups on systolic and diastolic pressure ratio, and hearth rate ratio ^#^.

	DTI	SI	^¶^ z
Systolic pressure ratio at the 1st session	1 (0.95–1.07)	1 (0.94–1.08)	−0.069
Systolic pressure ratio at the 30th session	1 (0.95–1.08)	1 (0.94–1.08)	−1.180
Diastolic pressure ratio at the 1st session	1 (0.97–1.07)	1 (0.93–1.11)	−0.687
Diastolic pressure ratio at the 30th session	1 (0.93–1.07)	1 (0.92–1.05)	−0.661
Heart rate ratio at the 1st session	1 (0.94–1.09)	1 (0.93–1.04)	−1.147
Heart rate ratio at the 30th session	1 (0.97–1.11)	0.98 (0.93–1.05)	−1.946

Values denote median (iqr). ^#^ A ratio between immediately before versus ‘after 15 min’ was calculated for the first and 30th session of intervention. ^¶^ z value derived from the Mann–Whitney test.

**Table 4:** Within-subject comparisons on cortisol levels by groups at pre- and post-intervention.

			Post-intervention		
			Irrelevant ^#^	Effective ^¥^	Negative ^¶^
	Pre-intervention	Irrelevant ^#^	2 (28.6)	2 (22.2)	0 (0)
DTI (*n* = 19)		Effective ^¥^	3 (42.9)	6 (66.7)	3 (100)
		Negative ^¶^	2 (28.6)	1 (11.1)	0 (0)
	Pre-intervention	Irrelevant ^#^	4 (36.4)	2 (25)	2 (28.6)
SI (*n* = 26)		Effective ^¥^	3 (27.3)	6 (75)	4 (57.1)
		Negative ^¶^	4 (36.4)	0 (0)	1 (14.3)

Values denote frequency with percentages in parenthesis. ^#^ Irrelevant = ratio between ‘immediately before’ versus ‘after 15 minutes’ calculated for the first and 30th session: a ratio of one meant that the cortisol concentration, and therefore the stress level (both low and high), had not changed. ^¥^ Effective = ratio between ‘immediately before’ versus ‘after 15 min’ calculated for the first and 30th session: if the ratio was greater than one, the cortisol concentration, and therefore the stress level, was decreased. ^¶^ Negative = ratio between ‘immediately before’ versus ‘after 15 minutes’ calculated for the first and 30th session: if the ratio was less than one, the cortisol concentration, and therefore the stress level, was increased.

## Data Availability

The data presented in this study are available on request from the first author.

## Abbreviations

NPT = non-pharmacological therapies; PWD = people with dementia; BPSD = behavioural and psychological symptoms of dementia; NPI-NH = Neuropsychiatric Inventory-Nursing Home; DT = doll therapy; NH = nursing home; DTI = doll therapy intervention; SI = sham intervention; ANCOVA = analysis of covariance; SE = standard error.
